# Changes in physico-mechanical properties of water caltrop fruit (*Trapa natans* L.) during the drying process

**DOI:** 10.1007/s00114-021-01768-4

**Published:** 2021-10-25

**Authors:** Cezary Toma, Mariusz Kukliński, Zygmunt Dajdok

**Affiliations:** 1 Department of Carpology, Kazimierz Wielki University, Chodkiewicza 30, 85-064 Bydgoszcz, Poland; 2 Department of Mechanics and Computer Methods, University of Science and Technology, Al. prof. S. Kaliskiego 7, 85-796 Bydgoszcz, Poland; 3 Department of Botany, University of Wrocław, Kanonia 6/8, 50-328 Wrocław, Poland

**Keywords:** Fruit, Trapa, Pericarp, Mechanical resistance, Hydration

## Abstract

The cosmopolitan water caltrop plant (*Trapa natans* L.) produces nuts, which in the maturing process develop very hard pericarps. This hardness, together with structure and shape (external spikes) of pericarp and seed, and the water contained in the fruit are responsible for their mechanical properties. This study determined the force needed to break *Trapa natans* nuts at various drying stages, with tests having been carried out at weekly intervals until the fruit dried completely. The amount of force necessary for cracking nuts at each of the 6 drying stages was determined, as well as the work of crushing calculated until the greatest compressive force (crushing force) was reached. The force needed to rupture the hydrated fruit in the horizontal plane was higher than that necessary for the rupture of dried fruit. The experiment showed that the maximum force needed to crush the fruit was 828.7 N and occurred when crushing the fruit after 2 weeks of drying, while the largest calculated crushing work was 2185.5 mJ for the same fruit. Other strength parameters were introduced to characterize mechanical properties of water caltrop in a more extensive scope. These are hardness defined as a ratio of compressive force increment to strain increment, specific crushing energy defined as a ratio of crushing work to water caltrop’s mass, and unit crashing force defined as a ratio of crushing force to caltrop’s thickness. All these parameters reached their highest mean values for pericarps after 2 weeks of desiccation. Mass measurements were also applied in modelling the desiccation process by the exponential function. The very dense pericarp material, after reaching maturity, slightly changes during drying. It can be used industrially as an extremely durable and biodegradable biological material. Results also suggest that the great evolutionary success of the species may result from the ability of the pericarp to protect its seeds, leading to the spread of this species in aquatic environments.

## Introduction

*Trapa natans* L., also known by its common name, water caltrop, is a cosmopolitan element of vascular flora (Zając and Zając 2009). The area of its natural occurrence covers Europe — from Spain in the west to Russia in the east and from the southern outskirts of the Scandinavian Peninsula in the north to the Balkan Peninsula and the Black Sea regions in the south (Hultén and Fries 1986; Piórecki 2014). Outside Europe, it occurs in Asia and Africa and as an invasive species in Australia and North America. Nowadays the *Trapa* genus belongs to the subfamily Trapoideae, the family Lythraceae, and the order Myrtales (Stevens 2001). The diversity of the size and shape of *Trapa natans* fruit has become the basis for distinguishing many taxa within the genus *Trapa*. Water caltrop is an annual aquatic plant rooted at the bottom of water bodies or rivers. Its shoots grow up to the water surface, on which they form leaf rosettes with flowers and fruit. The species occurs in stagnant and slow-flowing waters, mainly in oxbow lakes and fish ponds. In river beds, individuals of *Trapa natans* are found mainly along the banks, e.g. in bays between artificially built spurs. This species prefers eutrophic waters rich in nutrients. *Trapa natans* usually produces mono-seeded nuts 2–4.5 cm long and 1–3 cm wide. *Trapa natans* seeds are characterized by a high content of starch and protein. For this reason, they have long been used in Europe and Asia for human food and feed for farm animals (Piórecki 1980). The plant’s fruit is also used for medicinal and cosmetic purposes, among others (Chaudhary et al. 2012). This species is also used as a bioindicator of metals in lakes (Petrović et al. 2016). The pericarp of the fruit of this species is used as an organic herbicide (Javed et al. 2016). The chemical composition and pharmacological activity of pericarps of *Trapa natan*s have been studied, among others by Gani et al. (2010), Shalabh et al. (2012), and Bharthi et al. (2015). Additionally, studies on the physicochemical properties during *Trapa natans* nut storage were conducted by Singh et al. (2010). *Trapa natans* nuts are eaten by many animal species. The literature on *Trapa natans* nut consumption mainly mentions rodents, including muskrats (*Ondatra zibethicus* L.) (Muenscher 1937; Winne 1950; Kiviat 1993), beavers (*Castor canadensis* Kuhl), Norway rats (*Rattus norvegicus* Berkenhout), Eastern chipmunks (*Tamias striatus* Richardson), gray squirrels (*Sciurus carolinensis* Ord), and red squirrels (*Tamiasciurus hudsonicus* Bangs) (Hummel and Kiviat 2004). In one study, 58 intervertebrate species were associated with *Trapa* plants (Les 2018).

Materials published concerning the biology and ecology of *Trapa natans* and the features of its fruit lack data on the mechanical properties of the fruit. Examination of *Trapa natans* fruit in terms of mechanical properties will help to better understand the mechanisms of functioning and the importance of the fruit’s pericarp in the process of spreading this species and ensuring the best protection of its seeds against rodents. The fruit of the aquatic plant was used in the research, the pericarps of which are very succinct and may constitute a potential material for industrial use. The primary purpose of the research was to determine the amount of fruit crushing force during the drying process and the loss of water from both the pericarp and seeds. This work is innovative because the resistance of *Trapa natans* to crushing at various stages of drying is tested. So far, such studies have not been conducted on *Trapa natans* nuts.

Studying the physical and mechanical properties of agricultural products has been the subject of criticism and discussion for many years already and has attracted the attention of many researchers. The physical and mechanical properties of agriculture products are the most important parameters in the design of agricultural machinery sorting systems, transmissions, processing, and packaging systems (Ahangarnezhad et al 2019).

Research on the physical and mechanical properties of agricultural crops is related among others to Cassava harvesting machines (Atsyo et al. 2020); mechanization of the potato harvest (Ahangarnezhad et al. 2019); the possibility of breaking the seed shell of peanuts (Kurt and Arioglu 2018); the possibilities of red beet processing (Schäfer et al. 2020); designing equipment for transporting and storing peaches (Zohrabi et al 2013); transporting cherries (Sharifi-Sangdeh and Aghkhani 2018); distinguishing varieties of lemons (Baradaran Motie et al. 2014); and collecting data of apples for the harvesting robot (Li et al. 2017).

## Materials and methods

*Trapa natans* fruit collection was carried out in the third week of July 2018. It was collected from an Odra oxbow lake called Matunin, near Jelcz-Laskowice in Poland (N: 51° 0′ 47″; E: 17° 18′ 55″). The population in this reservoir is one of the largest in the region of Lower Silesia (SW Poland) and annually covers about 2 ha. The oxbow lake belongs to the typical eutrophic reservoir type with phytocoenoses of reed beds of the *Phragmitetea* class, communities of aquatic plants (*Potametea* class), and pleustonic (*Lemnetea minoris* class) plant communities (Kazuń 2005).

Due to the protection of *Trapa natans* in Poland, fruit collection was done after obtaining permission from the Regional Director for Environmental Protection in Wrocław (decision No. WPN.6400.22.2018.IL). Fruits were obtained directly from plants rooted at the bottom of the reservoir. Only ripe fruits were chosen, i.e. easily detached from the peduncle submerged in water. After harvesting, the fruits were placed in a sealed container (without air access) filled with water from the oxbow lake and delivered to the laboratory of the Department of Carpology at the University of Kazimierz Wielki in Bydgoszcz, where they were stored at 20 °C. The fruits were first pulled out of the water and placed in cotton gauze bags. The bags were then hung on a boom in a fume hood where the fruit dried naturally at 20 °C and relative humidity of 30%. Once a week, 5 fruits were selected from this collection for endurance tests. A total of 30 such trials were carried out using 30 fruits. The criteria for classifying ripe/unripe fruit was the size of the fruit. Morphological and anatomical observations of large and small fruit showed that the smaller the fruit, the less developed its pericarp, the larger the fruit, the thicker is the pericarp.

**Fig. 1:**
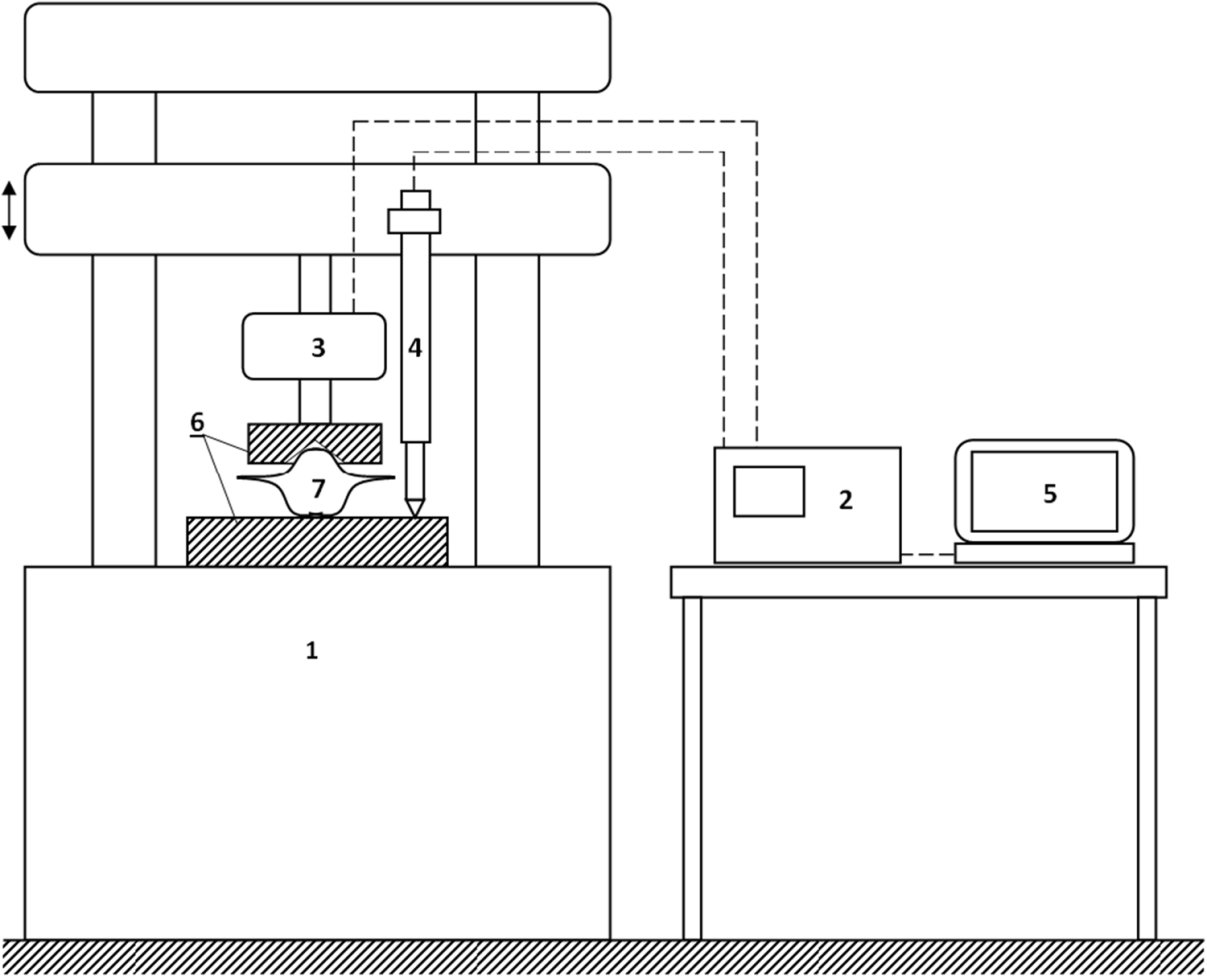
Schematic of the test stand for compressive loading: (1) ZD40 device, (2) MGCPlus measuring amplifier, (3) U2B force meter, (4) WA50 displacement meter, (5) computer, (6) clumps, (7) water caltrop

Nut crushing was carried out at the Department of Mechanics and Computer Methods of the University of Science and Technology in Bydgoszcz on a ZD40 device with an MGCPlus measuring amplifier, U2B force meter, and WA50 displacement meter. All measuring instruments have an accuracy class of 0.1%. The schematic of the test stand for compressive loading is shown in Fig. 1. Fruit crushing was carried out along the longitudinal axis. The tests were carried out on five samples after each period of desiccation: immediately out of the water and after 1 to 5 weeks after removal from the water. After rejecting the most extreme values, the rest of the results were subjected to further analysis.

According to statistical criteria, three samples constitute a scarce population; therefore instead of a standard error of the mean, the confidence interval for a level of confidence of 0.95 was calculated.

The crushing force (CF) is the maximum compressive force that occurs during each crushing test. The crushing work (CW) was calculated numerically from the starting point of the test until the CF was reached, as given in the subsequent Eq. (1):

1 $${CW}=\int_0^{{ Z}\max} {Fdz}$$

where *F* is an instant applied compressive force and *z*_max_ is the deformation of the water caltrop measured in the direction of the force *F* that occurs when *F* reaches its maximum value (CF).

In the literature, one can find attempts to calculate stresses in the nut’s shell and its Young’s modulus approximating the nut’s pericarp by a regular sphere and applying the equations of shell theory. For example, according to Wang and Mai (1995), such a method was applied to macadamia nuts. However, in contrast to macadamia nuts, water caltrops have a very irregular pericarp, so it is challenging to estimate the stresses that occur in them during compressive loading. Nevertheless, a hardness parameter (HRD) was introduced to reflect the relationship between compressive loading (N) and longitudinal strain (mm/mm). The hardness parameter is defined by Eq. (2):

2 $$HRD=\frac{{\Delta F}_C}{\Delta_\varepsilon}\left[\frac N{mm/mm}\right],$$

where Δ*ε* is longitudinal strain increment defined as a ratio of linear deformation increment to initial length (LZ) of the water caltrop, which relates to the respective compressive force increment Δ*F*_*C*_. Eventually, the introduced parameter HRD $\left[\frac N{mm/mm}\right]$ has a similar meaning to elasticity modulus $\left[\frac{MPa}{mm/mm}\right]$.

The fruit’s pericarps were examined with a Hitachi scanning electron microscope at the Silesian University of Technology in Katowice. The outer and inner surfaces of the pericarp and a cross-section of the pericarp were observed.

## Results

### Structure of the *Trapa natans* fruiting plant in SEM

**Fig. 2:**
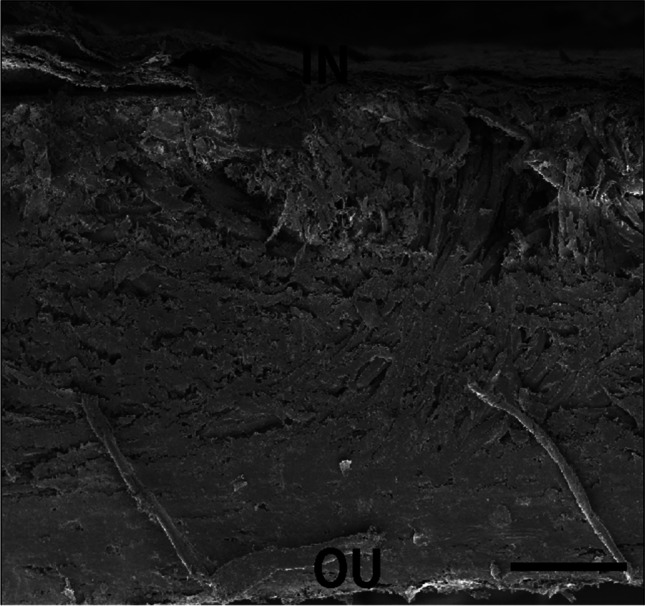
Cross-section of the wall of the *Trapa natans* fruit in SEM. OU, outer surface; IN, internal surface. Bar, 300 μm

**Fig. 3:**
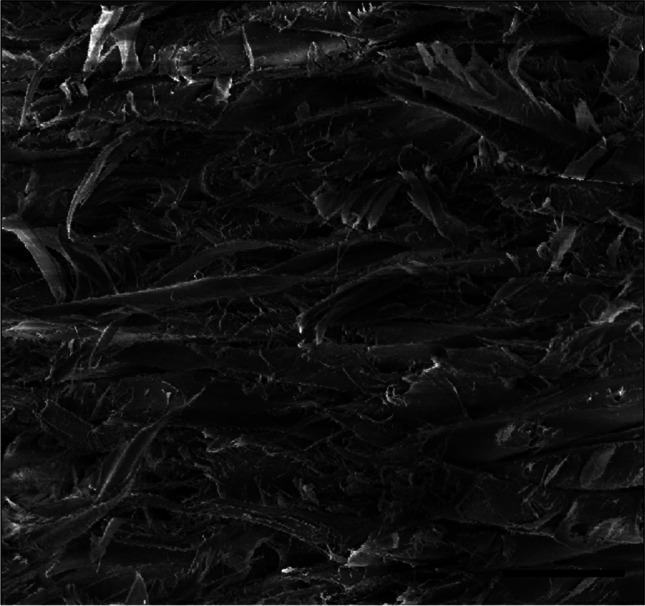
Breakthrough wall of *Trapa natans* fruit. Bar, 60 μm

The cross-section of the *Trapa natans* fruit wall, 1327–1420 μm thick, has no uniform structure. The examined fragment of the pericarp comes from the central part of the fruit. The outer part of the pericarp is more compact, 500 μm thick with densely arranged fibres, while the inner part of 900 μm thick is less compact (Fig. 2). Breaking through the fruit wall shows the end of a wood fibre pericarp with a thickness of 23–28 μm (Fig. 3).

**Fig. 4:**
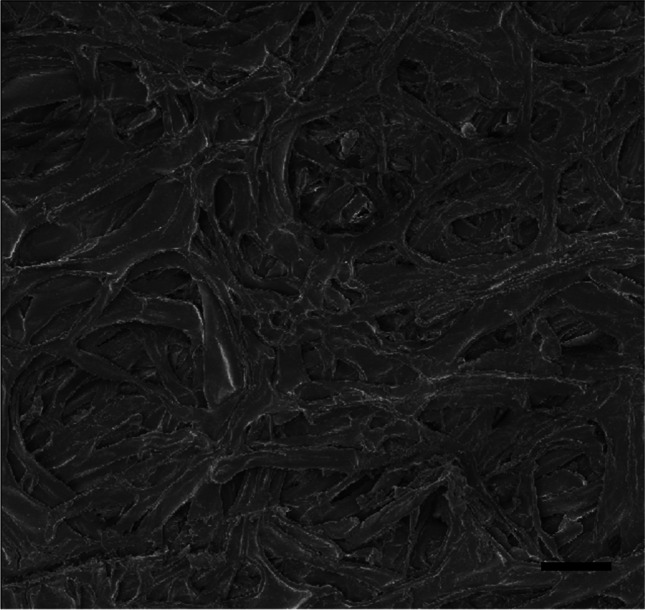
Internal structure of the fruit wall of *Trapa natans*. Bar, 100 μm

**Fig. 5:**
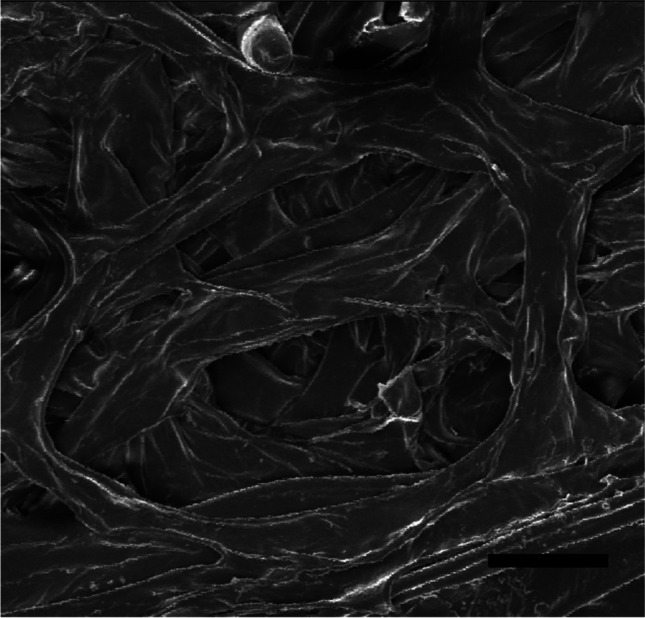
Internal structure of the fruit wall of *Trapa natans*. Bar, 50 μm

Using a scanning electron microscope, very densely arranged wood fibres form a spatial network. The fibres arranged in the inner layer of the pericarp are very regular and intertwine with each other to form a structure resembling a composite. Cavities are 55–214 μm in size and are arranged at different depths in the pericarp (Figs. 4–5). The pericarp of *Trapa natan*s has such a compact structure that it is difficult to cut it with a scalpel to obtain a cross-section.

### Pericarp strength tests

**Fig. 6:**
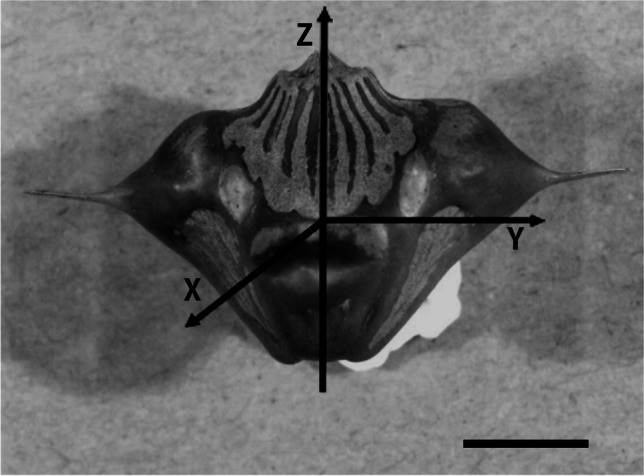
Example water caltrop and assumed axes of Cartesian coordinate system with a scale given in centimetres. Bar, 1 cm

**Fig. 7:**
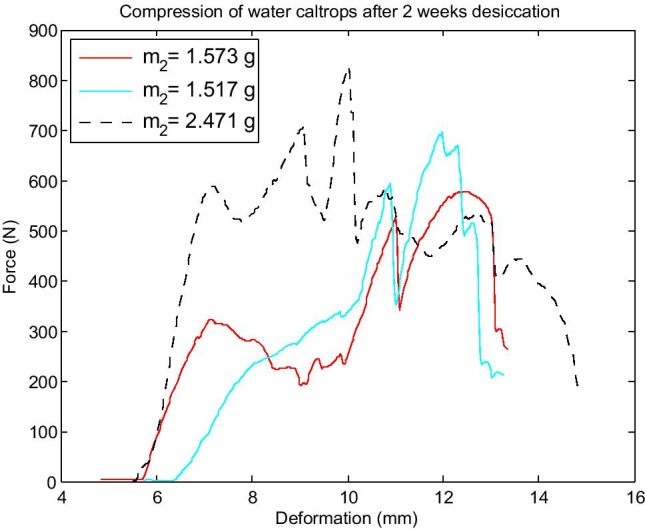
The course of compressive force as a function of deformation for three samples of given masses after 2 weeks desiccation

**Fig. 8:**
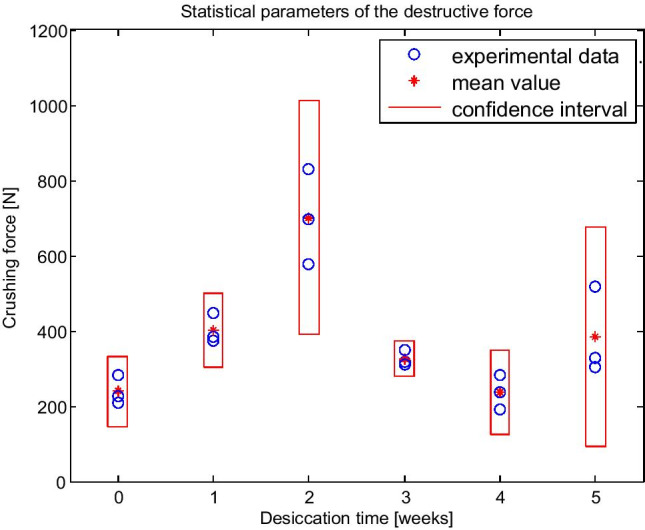
Visualization of measurements and statistical parameters of crushing force after each period of desiccation (in weeks)

The force was applied along axis *Z* during the compression tests, as seen in Fig. 6. An example graph in Fig. 7 presents the course of compressive force as a function of deformation along axis *Z* for three samples after 2 weeks of desiccation. These are sample numbers 7–9 in Table 1. The measured values of masses, crushing forces, and crushing works are also given in Table 1. Figure 8 shows a visualization of the measured values of CF after each period of desiccation, their mean values, and the limits of confidence intervals.

**Table 1:** Mechanical properties of water caltrops after each week of desiccation: *LZ*, initial length of water caltrop; *TWS*, thickness measured without spikes; *CF*, crushing force; *HRD*, hardness parameter expressing the relationship between compressive loading and longitudinal strain

Spec no.	Mass after number of weeks of desiccation (g)									
	*m*_0_	*m*_1_	*m*_2_	*m*_3_	*m*_4_	*m*_5_	LZ (mm)	TWS (mm)	CF (N)	HRD (N)
	out of water	1 week	2 weeks	3 weeks	4 weeks	5 weeks				
1	3.508						20.16	10.10	209.7	6171
2	3.803						20.13	10.30	281.9	2545
3	5.647						20.46	10.36	227.9	4365
4	3.916	2.426					20.16	10.25	372.0	3071
5	3.516	2.395					20.25	10.32	385.9	6874
6	4.24	2.322					20.41	10.15	446.1	8017
7	3.413	2.546	1.573				20.22	10.22	578.7	4995
8	3.016	2.379	1.517				20.08	10.33	698.8	7293
9	4.679	3.381	2.471				20.22	10.30	828.7	10.443
10	3.676	2.246	1.735	1.333			20.14	10.22	317.6	4220
11	4.081	1.641	1.494	0.984			20.20	10.35	311.7	4474
12	4.258	3.041	2.562	2.479			20.35	10.31	347.6	4395
13	3.009	1.162	0.981	0.964	0.940		20.93	10.11	283.4	3937
14	3.887	2.602	1.237	1.049	0.978		20.11	10.33	192.6	2236
15	3.352	2.106	1.168	1.087	1.077		20.94	10.25	237.8	1771
16	3.111	2.283	1.668	1.662	1.624	1.544	20.10	10.41	302.7	7120
17	4.407	3.232	2.398	2.25	2.213	2.031	20.27	10.30	517.8	7754
18	3.322	2.089	1.814	1.697	1.523	1.248	20.15	10.20	329.7	4492

The analysis of results given in Table 1 and Fig. 8 reveals that after 2 weeks of desiccation, the structure of the water caltrop is the most resistant and the greatest forces are needed to crush the sample. The maximum crushing force registered during tests was 828.7 N and the largest calculated crushing work was 2185.5 mJ for the same fruit number 9 in Table 1. The course of the crushing test for this sample is visualized by the dashed line in Fig. 7. Nevertheless, the measurements of the CF after 2 weeks of desiccation have the widest confidence intervals because of the significant influence of internal structure on the mechanical properties of the water caltrop. The structure is highly anisotropic, as seen in Fig. 3.

The hardness parameter (HRD) was defined as a ratio of the compressive force increment to the longitudinal strain increment. These increments were measured in the first, steepest linear growth of the compressive force as a function of deformation. For example, in Fig. 7, the hardness of the nut with a mass *m*_2_ = 1.517 g was calculated in a range of displacement from 10.28 to 10.75 mm. Irregularities in the initial stage of each line in Fig. 7 reflect possible slight movements of the sample in compressing clumps.

Table 1 presents the geometrical and mechanical properties of water caltrops used for compressive testing. Two other parameters were calculated to consider the influence of mass and size on the caltrop’s compression strength. These were the specific crushing energy (SCE) and the unit crushing force (UCF). SCE is defined as a ratio of crushing work to water caltrop’s mass, and UCF is a ratio of crushing force to its thickness (TWS) measured along the *X* axis (Fig. 6) without spikes. Parameters LZ and TWS are nut’s dimensions that can be precisely measured. Wide confidence intervals of all strength parameters, i.e. CF, HRD, SCE, and UCF, indicate that the pericarp’s irregularity and anisotropic shell structure predominantly influence the mechanical properties of water caltrops. Nevertheless, the analysis of results in Table 1 and Table 2 concludes that after 2 weeks of desiccation, all the nut’s strength parameters tend to their maximum values.

**Table 2:** The mean values of physical and calculated compression parameters and their confidence intervals: *LZ*, initial length of water caltrop; *TWS*, thickness measured without spikes; *CF*, crushing force; *CW*, crushing work; *HRD*, hardness parameter expressing the relationship between compressive loading and longitudinal strain; *SCE*, specific crushing energy; *UCF*, unit crushing force

Desiccation time	Right out of water	1 week	2 weeks	3 weeks	4 weeks	5 weeks
Mass (g)	4.319 ± 2.880	2.381 ± 0.133	1.875 ± 1.33	1.600 ± 1.943	0.998 ± 0.176	1.608 ± 0.982
LZ (mm)	20.25 ± 0.45	20.27 ± 0.31	20.17 ± 0.20	20.23 ± 0.27	20.66 ± 1.18	20.17 ± 0.22
TWS (mm)	10.28 ± 0.21	10.24 ± 0.21	10.28 ± 0.14	10.29 ± 0.17	10.23 ± 0.28	10.30 ± 0.26
CF (N)	240 ± 93	401 ± 98	701 ± 311	326 ± 48	238 ± 113	383 ± 291
CW (mJ)	276 ± 406	1058 ± 699	2034 ± 550	565 ± 839	375 ± 119	428 ± 197
HRD (N)	4360 ± 4505	5988 ± 6432	7577 ± 6795	4363 ± 323	2648 ± 2833	6455 ± 4296
SCE (mJ/g)	68 ± 116	444 ± 275	1138 ± 593	461 ± 1095	375 ± 82	270 ± 76
UCF (N/mm)	23.3 ± 3.58	39.2 ± 10.3	68.2 ± 29.6	31.6 ± 4.64	23.3 ± 11.7	37.2 ± 28.4

The measured masses of caltrops were also applied to model the desiccation process by an exponential curve given by function (3):

3 $$m={B+(m_{\mathbf0}-B)e}^{-t},$$

where *B* is the constant calculated by the least square method.

**Fig. 9:**
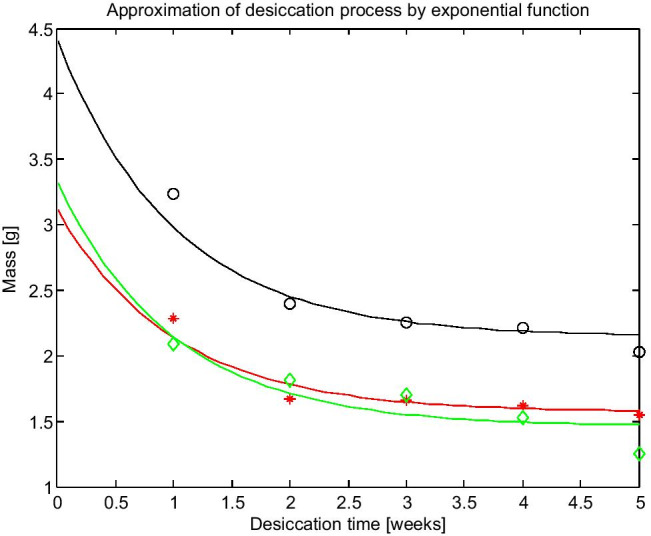
Comparison of measured masses during the desiccation process and their approximation by the exponential function (3)

A graph comparing the measured values of caltrop mass after each week of desiccation with the values calculated by function (3) is presented in Fig. 9.

## Discussion

*Trapa natans* nuts reduced their weight during drying in laboratory conditions. However, this process did not increase the mechanical resistance of the nuts. In the natural environment, *Trapa natans* fruits are found in water, on the outskirts of water reservoirs and in bottom sediments, e.g. fish ponds, where they retain their germination potential for many years. However, the subject of research was not germination, depending on many factors, but the persistence of the pericarp, i.e. its resistance to crushing force during the drying process. Lower mechanical resistance of the pericarp of *Trapa natans* occurs in ripening nuts that have not yet reached the final dimensions for the species.

This study showed that a mature pericarp has the highest mechanical resistance when its hydration is still relatively high (second week of drying). Therefore, as long as the *Trapa natans* fruits are moist, their resistance to destruction during biting by animals is the highest. This feature indicates *Trapa’s* significant evolutionary adaptation to protect its seeds in aquatic environments.

If we look at the structure of the *Trapa natans* nut pericarp, it becomes clear that it supports nut crack resistance due to its compact composite structure. It is worth adding that nuts are biological objects that are characterized by variability. One symptom of this variation is the different degrees of pericarp thickness in different locations of the pericarp. The fruit’s complete resistance to cracking depends on many fruit features and external factors related to the species’ environment, such as water quality and temperature. Among the fruit features affecting the fruit’s total mechanical resistance are the size and shape of the fruit, the degree of fruit spike formation, pericarp thickness, and the degree of tissue hydration. It would also be interesting to examine the resistance of the pericarp to crushing at various temperatures. This characteristic of pericarp fibres may be of great importance in industrial use and requires further research.

Natural fibres are usually believed to have several benefits compared to synthetic fibres, such as availability, low cost, low density, acceptable modulus-weight ratio, high acoustic damping, low manufacturing energy consumption, low carbon footprint, and biodegradability (Mohanty et al. 2005). Plant fibres can also be regarded as fillers to replace the more expensive polymers and improve the green credentials of the final composite parts (Fortea-Verdejo et al. 2017). For example, fibres from fruit produced by species of the genera *Borassus* and *Tamarind* have been successfully used in composites used in construction engineering (Nayak et al. 2019).

Among the fibres derived from *Areca catechu*, it is worth paying attention to the construction of the betel nut and glass fibre composite. Palm fruit fibres can be successfully used as reinforcing fibres in polyethylene composites in the automotive industry and food packaging (Merajul Haque and Hasan 2016). Natural fibre composites are considered to have potential use as reinforcing material in polymer matrix composites because of their good strength, stiffness, low cost, environmental friendliness, and biodegradability (Srinivasa and Bharath 2011). Natural fibres are playing an increasing role as reinforcement in polymer composites (Bensadoun et al. 2017). The need for natural fibre-reinforced composites is increasing at a very fast rate because of their ecofriendly production, decomposition, high specific strength, abundance, and good physical and mechanical properties (Latif et al. 2019). Palm fruit fibres and sawdust are used as building materials (Sosu et al. 2011). Palm fibres are also used in water filtration filters (Idris et al. 2017), for the production of bio-oil (Onifade et al. 2017), frictional material (Achebe et al. 2019), and in biomedicine (Namvar et al. 2014). Up to now are no reports regarding the mechanical properties of *Trapa natans* nuts. Existing reports relate to the physical and mechanical properties of terrestrial nuts that are used industrially. In *Juglans regia* (Ercisli et al. 2011), features such as length, width, thickness, mass, geometric mean diameter, deformation at cracking, and cracking force have been examined. The microstructure and mechanical properties of *Macadamia* nutshells (Wang and Mai 1995) have been examined and heat treated. *Macadamia* nutshells are similar to wood, except that they may have different aspect ratios. The strength of the *Macadamia* nutshell is naturally a compromise between attaining high strength and achieving isotropy as required by the need to provide all-round protection to the seed (Wang and Mai 1995). In the case of *Trapa natans*, the seed does not adhere completely to the pericarp, as is the case with *Macadamia* nuts, which affects the overall mechanical resistance of the nut. In addition, the seed of the aquatic plant is dehydrated and shrinks in the drying process, which causes the seed to stand slightly away from the pericarp wall in the dried fruit.

Comparing compression parameters of water caltrop with the mechanical properties of other types of nuts available in the scientific literature leads to the following conclusion: water caltrops need a greater compressive force and work to be crushed than walnuts tested (Ercisli et al. 2011). Nevertheless, the water caltrop’s compressive strength is lower than that of macadamia nuts (Wang and Mai 1995).

The mechanical properties of pistachios increased with decreasing moisture. Deformation and deformation ratio, which decreased with decreasing moisture content, were also studied (Razavi and Edalatian 2012). In the case of *Trapa natans* nuts, they had the greatest mechanical resistance to compression during the 2nd week of testing, i.e. when they were not completely dried. The force in newtons needed to crush the *Trapa natans* nut was 25 × greater than the maximum force used to crush the pistachio nut.

With the recent development of biomimetics (Vincent 2012) as a method of incorporating the characteristics of biological materials and microstructures into engineering design, more and more attention is being paid to the direction of biological materials research. We hope that initial research into the structure and mechanics of the *Trapa natans* pericarp will stimulate scientists’ interest in further studies on the properties of the fibre and its use for industrial purposes.

Research has shown that the fruit is the most durable in the second week of drying. This is of great practical importance when looking for biodegradable material with high crush resistance. Further testing of *Trapa natans* pericarps should be taken from the nuts in the 2nd week of drying. An important evolutionary significance of our research is determining the maximum hardness of fruits that are still partially hydrated and as resistant to animal chewing as possible. *Trapa natans* fruits protect the seeds from eating to the maximum extent as long as they are moist, so as long as they retain their ability to germinate.

## Conclusions

The study showed the effect of water content on water caltrop fruit resistance to crushing and resistance to fruit destruction by animals. Different degrees of hydration significantly impact the amount of force that causes the fruit to crack. Greater force is needed for crushing hydrated fruit than for crushing dry fruit. As the water content in the pericarp increases, the force needed to break the fruit increases. Thanks to the high strength of the pericarp, the seeds are well protected against mechanical damage, including cracking, and complete drying, which, among other things, allows them to be transported through water or by animals over long distances. The mechanical properties of *Trapa natans* fruit and pericarp building fibres described in the article indicate their potential for application in the industry. Potentially, the plant material of *Trapa natans* pericarps, due to its high mechanical resistance, can be used in industrial processing, e.g. the production of seals for amphibious vehicles, hovercraft, inflatable boats, and rafts for recreational purposes or small reinforcing elements in aquarium systems or in the production of fishing equipment.

## Abbreviations

*B*: The constant parameter in the desiccation exponential function calculated by the least square method
CF: Crushing force (N)
CW: Crushing work (mJ)
HRD: Hardness parameter expressing the relationship between compressive loading (N) and longitudinal strain (mm/mm)
IN: Internal surface
LZ: Initial length of water caltrop in the direction of axis *Z* (mm)
*m*_*t*_: Mass of water caltrop after “*t*” weeks of desiccation (g)
*m*_0_: Mass of the water caltrop right out of water (g)
OU: Outer surface
SCE: Specific crushing energy (mJ/g)
TWS: Thickness measured along the *X* axis without spikes (mm)
UCF: Unit crushing force (N/mm) UCF = CF/TWS
